# Effects of obesity on NK cells in a mouse model of postmenopausal breast cancer

**DOI:** 10.1038/s41598-020-76906-5

**Published:** 2020-11-26

**Authors:** Julia Spielmann, Laura Mattheis, Juliane-Susanne Jung, Henrik Rauße, Markus Glaß, Ina Bähr, Dagmar Quandt, Jana Oswald, Heike Kielstein

**Affiliations:** 1 Institute of Anatomy and Cell Biology, Medical Faculty of Martin Luther University Halle-Wittenberg, Grosse Steinstrasse 52, 06108 Halle (Saale), Germany; 2 Deptartment of Internal Medicine I, Medical Faculty of Martin Luther University Halle-Wittenberg, Halle (Saale), Germany; 3 Clinic for Psychosomatics and Psychotherapy, Landschaftsverband Westfalen-Lippe Clinic, Lengerich, Germany; 4 Institute of Molecular Medicine, Charles Tanford Protein Center, Medical Faculty of Martin Luther University Halle-Wittenberg, Halle (Saale), Germany; 5 Regenerative Medicine Institute (REMEDI) at CÚRAM Centre for Research in Medical Devices, School of Medicine, College of Medicine, Nursing and Health Sciences, National University of Ireland, Galway, Ireland

**Keywords:** Cancer, Immunology, Oncology, Risk factors

## Abstract

Obesity is a widely spread disease and a crucial risk factor for malign disorders, including breast cancer of women in the postmenopause. Studies demonstrated that in case of obesity crucial natural killer (NK) cell functions like combating tumor cells are affected. This study aims to analyze NK cells and NK cell receptor expression of obese mice in a model for postmenopausal breast cancer. Therefore, female BALB/c mice were fed either a high fat or a standard diet. Thereafter, ovaries were ectomized and a syngeneic and orthotopical injection of 4T1-luc2 mouse mammary tumor cells into the mammary adipose tissue pad was performed. Obese mice showed increased body weights and visceral fat mass as well as increased levels of leptin and IL-6 in plasma. Moreover, compared to the lean littermates, tumor growth was increased and the NKp46-expression on circulating NK cells was decreased. Furthermore, the activating NK cell receptor NKG2D ligand (MULT1) expression was enhanced in adipose tissue of obese tumor bearing mice. The present study gives novel insights into gene expression of NK cell receptors in obesity and aims to promote possible links of the obesity-impaired NK cell physiology and the elevated breast cancer risk in obese women.

## Introduction

Obesity represents a major health problem in numerous countries, as the prevalence for obesity is continuously rising worldwide. Overweight and obesity among adults are defined as a body mass index (BMI) of 25.0 to 29.9 kg/m^2^ and 30 kg/m^2^ or more respectively^1^. Data of the World Health Organization (WHO) presented in detail, that in 2016 more than 1.9 billion adults were overweight and of these over 650 million were classified obese^2^. The association of obesity with many chronical conditions, such as cardiovascular diseases, type 2 diabetes mellitus as well as renal, musculoskeletal and psychological disorders is recognized^3–6^. Nevertheless, the susceptibility to infections is increased in obese individuals and overweight and obesity are discussed as crucial risk factors for a variety of cancer types, including esophageal, colorectal, gallbladder, pancreatic, liver, postmenopausal breast, ovarian, endometrial, kidney and prostate cancer^7,8^. For 2018, the German Cancer-Research Center estimated about 6.9% of all new cancer cases to be caused by overweight and obesity in Germany. Representing the most frequent cancer types in man and woman, this corresponds to 23.2% for colorectal and respectively to 16.2% for postmenopausal breast cancer of all new overweight or obesity related cancer cases^9^. Moreover, the Global Burden of Disease Study showed that four million deaths worldwide were caused by overweight and obesity in 2015^10^. Obese individuals with a BMI higher than 40 showed elevated death rates from all cancers that were 52% higher for men and 62% higher for woman compared to normal weights as revealed by a prospective study of Calle et al*.*^11^. Moreover, they estimated that 14% of all deaths from cancer in men and 20% of those in women could be accounted to overweight and obesity in the United States. Avoiding weight gain or losing weight by bariatric surgery can reduce cancer risk ^12,13^. Studies on the influence of obesity on survivorship of cancer patients showed worse effects on quality of life, cancer recurrence, cancer progression, prognosis and survival^11,14–16^. However, mechanisms underlying the increased cancer risk under obesity are still not fully understood and under investigation^17^. Next to insulin resistance, reduced physical activity and sex hormones, also the influence of obesity on immune cell functions is considered^18–21^. Previous studies showed that adipocytokines like leptin obviously have an impact on the immune system^22^. Taking a closer look, the functions of natural killer (NK) cells, which are an essential part of the innate immune system, represent 10 to 15% of the peripheral lymphocytes. It is known, that they are impaired by leptin, which is secreted by the adipocytes and increases relative to the gain of body fat mass^23,24^. NK cells play an important role in the early defense by identifying and killing virally infected and tumor cells without prior sensitization and restriction by major histocompatibility (MHC) antigens^25^. The identification of targets by NK cells is regulated by the expression of activating and inhibitory receptors and in particular by binding of ligands expressed by the target cell, followed by the lysis of target cells^26^. It has been shown that obese individuals show a dysfunction of NK cells^23,24,27–29^. Moreover, a prospective study demonstrated that impaired NK cell functions are associated with an increased cancer incidence^30^. Interestingly, obese patients who lost body weight and body fat mass by bariatric surgery or by exercise training and nutrition counseling could reverse their impaired NK cell activity and NK cell-mediated cytokine synthesis^31,32^. In addition, a recent study showed that lipid accumulation in NK cells from obese individuals is associated with the loss of NK cell cytotoxicity against tumor cells and could be restored by metabolic reprogramming^33^. This indicates that there might be a promising link between impaired NK cell functions in obese individuals and the increased risk for cancer under obesity.

The aim of the present study was to mimic postmenopausal breast cancer under diet-induced obese (DIO) conditions in a mouse model to investigate immune cell frequencies and associations between functional NK-cell marker and tumor development in obesity.

Hence, to induce obesity female BALB/c mice received a high-fat diet and to achieve a postmenopausal status ovariectomy was conducted. To induce a mammary carcinoma, triple negative 4T1-luc2 cells, that do not express the estrogen receptor (ER), progesterone receptor (PgR) or the gene for human epidermal growth factor receptor 2 (HER2), were injected orthotopically in the mammary fat pad. This enabled us to investigate the isolated effect of extragonadal derived estrogen on NK cells, independently of the known proliferating effect of estrogens on breast cancer cells. To investigate initial NK cell profiles, we analyzed NK cell numbers, NK cell receptor expression and cytokine-levels 20 h after application of the tumor cells (short-term experiment). Therefore, effects of diet-induced obesity on early tumor development in a mouse model for postmenopausal breast cancer could be studied. Furthermore, in the long-term experiment, tumor growth was monitored by bioluminescence imaging and NK cell receptor natural killer group 2 member D (NKG2D) as well as the ligands UL16-binding protein-like transcript 1 (MULT1) and retinoic acid early inducible-1 gene (Rae-1) were determined in adipose tissue of mice.

## Results

### Body and organ weights

**Figure 1:**
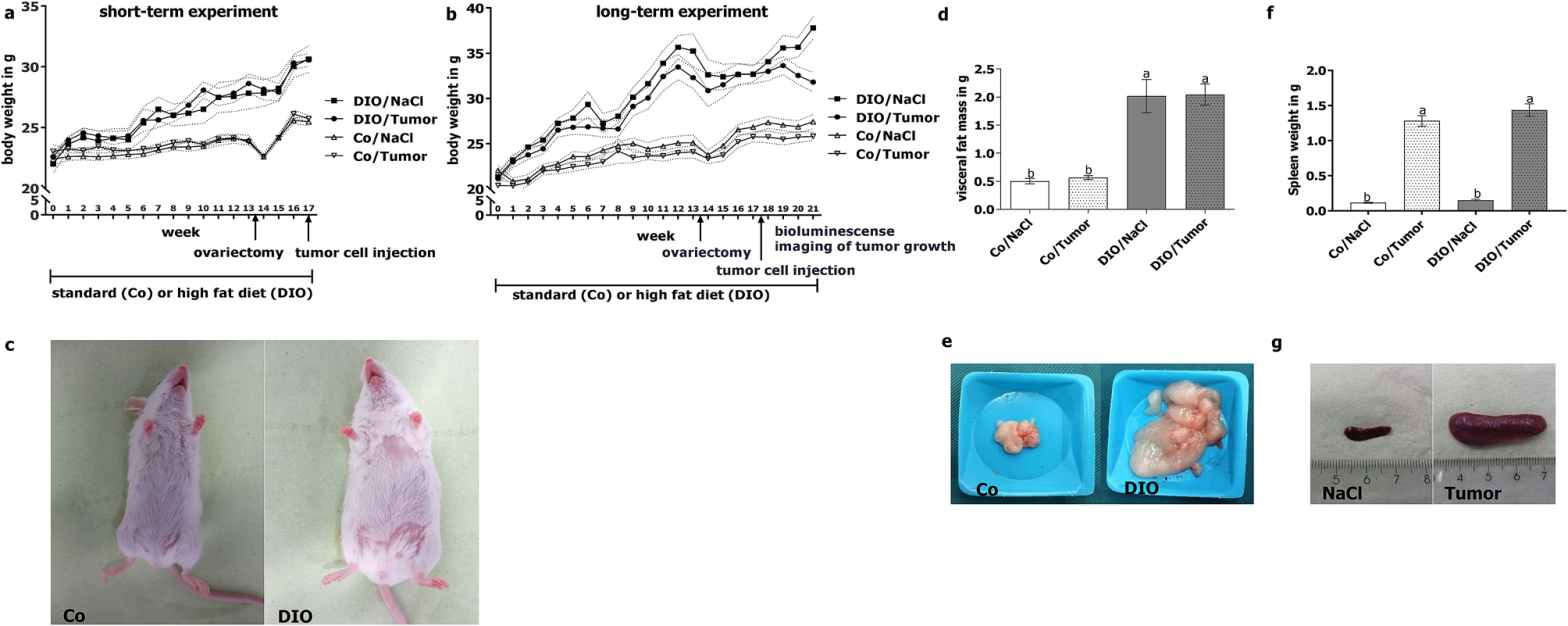
Experimental setting (**a**,**b**) and anthropometric data (**a**–**g**) of mice in short-term and long-term experiment. Weight gain of mice in response to high fat diet (DIO) compared to control diet (Co) and sodium chloride (NaCl) or 4T1-luc2 (Tumor) over a period of 17 weeks (short-term experiment, (**a**) or 21 weeks (long-term experiment, (**b**). Representative pictures for weight gain of mice of the Control or DIO group (**c**) and visceral fat mass of mice in the short-term experiment (**d**,**e**). Spleen weight of mice in the long-term experiment (**f**) and representative pictures (**g**) of spleen of 4T1-luc2-tumor-bearing mice compared to sodium chloride-injected mice (NaCl). Values represent means ± SEM, n = 7 mice/group. Different superscript letters (**a**,b) indicate significant differences between individual experimental groups analyzed by Tukey´s multiple comparison test (p ≤ 0.05).

Mice fed the DIO diet gained significantly more weight compared to mice fed the control diet (Fig. 1a–c). In the long-term experiment significant weight gain of DIO-fed animals compared to control diet fed animals even continued after 4T1-luc2 tumor-cell-injection and during tumor growth (Fig. 1b,c). In contrast to control diet fed animals, mice of the DIO-fed group lost weight under tumor development, though maintaining a higher body weight compared to mice of control diet group. Appropriately, in both experiments DIO-fed animals gained significantly more visceral fat mass compared to animals fed the control diet (Fig. 1d,e; Supplementary Table S1 and S2). Nevertheless, tumor growth during the long-term experiment led to a significantly loss of visceral fat mass in control diet as well as DIO-fed mice (Supplementary Table S2).

In the short-term experiment, mice of the DIO-fed group showed significantly increased spleen weights compared to control diet-fed animals (Supplementary Table S1). Tumor growth during the long-term experiment led to significantly higher spleen weights compared to animals receiving sodium chloride (NaCl) injection as a control (Fig. 1f,g; Supplementary Table S2). Liver weights of mice did not differ between groups of the short-term experiment (Supplementary Table S1). In the long-term experiment, mice of the DIO group and mice receiving tumor cells independent of the diet showed significantly increased liver weights compared to control diet-fed mice receiving NaCl injection (Supplementary Table S2).

### Plasma levels of leptin, IL-6, IFN-γ and TNF-α in short-term experiment

**Figure 2:**
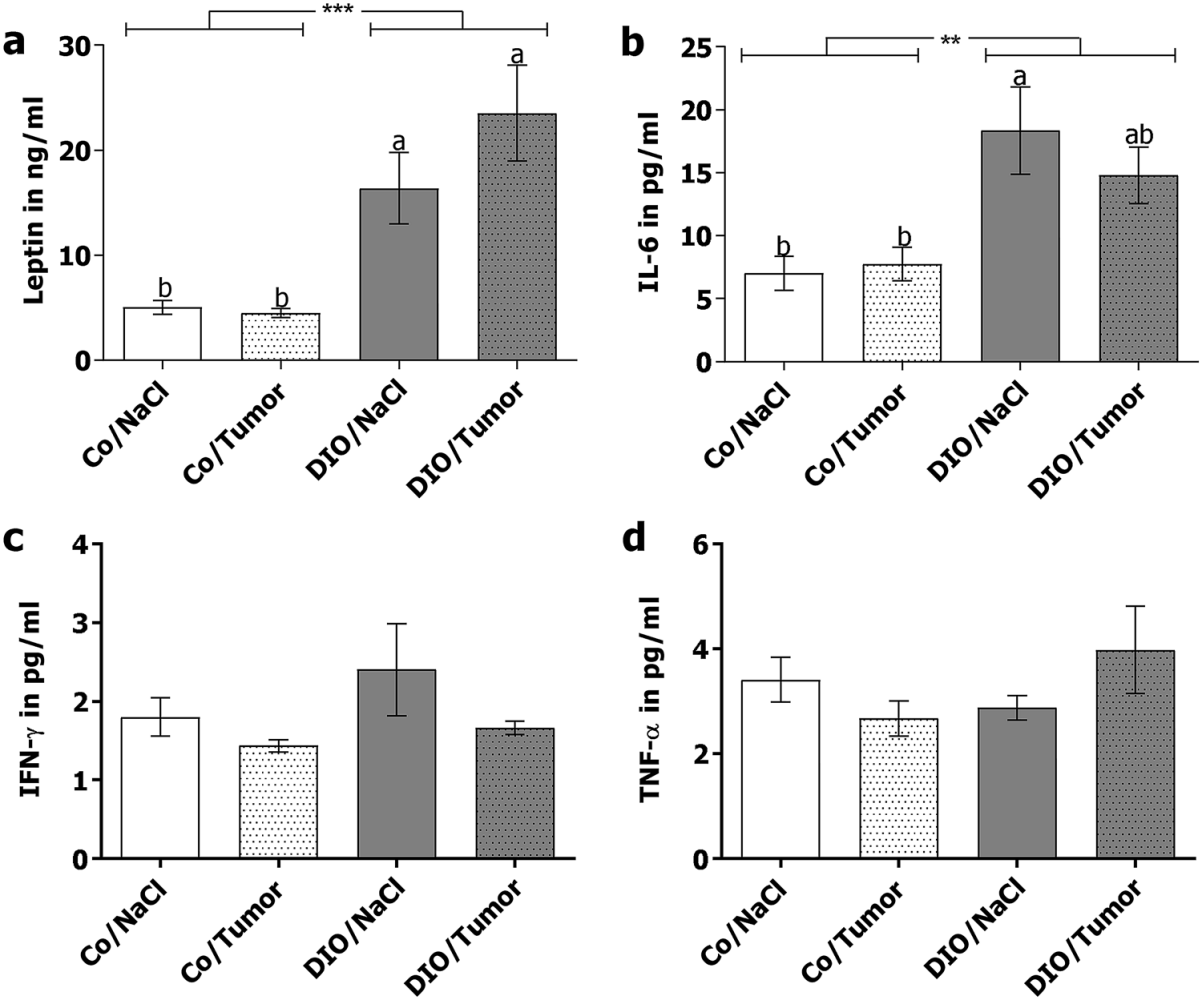
Plasma concentrations of leptin (a), IL-6 (b), IFN-γ (**c**) and TNF-α (**d**) of control diet-fed mice (Co) and DIO diet fed mice (DIO) receiving either sodium chloride (NaCl) or 4T1-luc2 breast cancer cells (Tumor) in short term experiment. Values represent means ± SEM, n = 7 mice/group. * indicates significant differences of means between mice receiving DIO diet (DIO) compared to mice receiving control diet (Co) analyzed by two-way ANOVA (** p ≤ 0.01; *** p ≤ 0.001). Different superscript letters (**a**,**b**) indicate significant differences between individual experimental groups analyzed by Tukey´s multiple comparison test (p ≤ 0.05).

Diet-induced obesity led to significantly increased leptin levels in the plasma of mice, compared to their corresponding control diet-fed groups (Fig. 2a). Plasma levels of interleukin (IL)-6 were increased in DIO-fed mice compared to mice fed the control diet. DIO-fed mice, which received NaCl-injection showed the highest IL-6 levels in the plasma and differed significantly to mice fed the control diet (Fig. 2b). Interferon (IFN)-γ- and tumor necrosis factor (TNF)-α-plasma levels did not differ between the four groups (Fig. 2c,d).

### Monitoring of tumor development by bioluminescence imaging and caliper measurement

**Figure 3:**
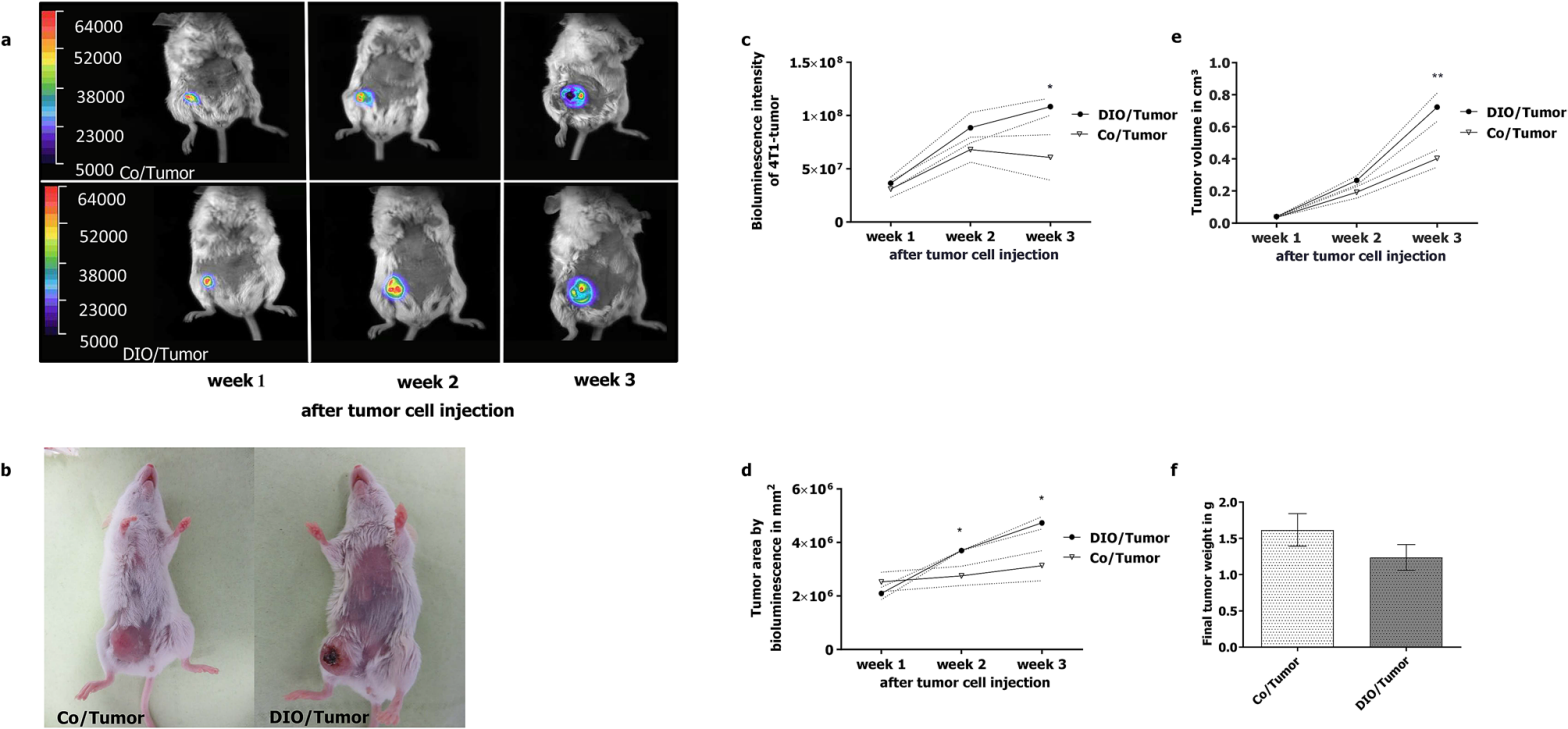
Characterization of tumors in mice of the long-term-experiment. Representative pictures for visual tumor development (**b**), for bioluminescence (**a**), bioluminescence intensity (**c**) and tumor area determined by bioluminescence area (**d**) of tumor-growth monitoring on day 7 (week 1), day 14 (week 2) and day 21 (week 3) after orthotopically injection of 10^6^ 4T1-luc2 breast cancer cells (Tumor) in control diet-fed (Co) compared to DIO diet-fed mice (DIO). Tumor volume was determined weekly by caliper measurement (**e**) and final tumor weight was documented (**f**) after sacrification of mice. * indicates significant differences of means between mice receiving DIO diet (DIO) compared to mice receiving control diet (Co) at the different time points analyzed by two-way ANOVA (* p ≤ 0.05; ** p ≤ 0.01).

Monitoring of tumor growth by bioluminescence imaging in the long-term experiment revealed that all mice, who received 4T1Luc2 cells developed a tumor and that mice fed the high-fat diet showed a significantly increased tumor area compared to mice fed the control diet from week two after tumor challenge on (Fig. 3a,b,d). Bioluminescence intensity and caliper measurement of the tumors were significantly higher in DIO-mice compared to control mice after three weeks of tumor growth (Fig. 3a–e). Nevertheless, tumor weight did not differ between control- and DIO-mice at the end of the long-term experiment (Fig. 3f).

### Flow cytometry analysis of peripheral blood immune cells

**Figure 4:**
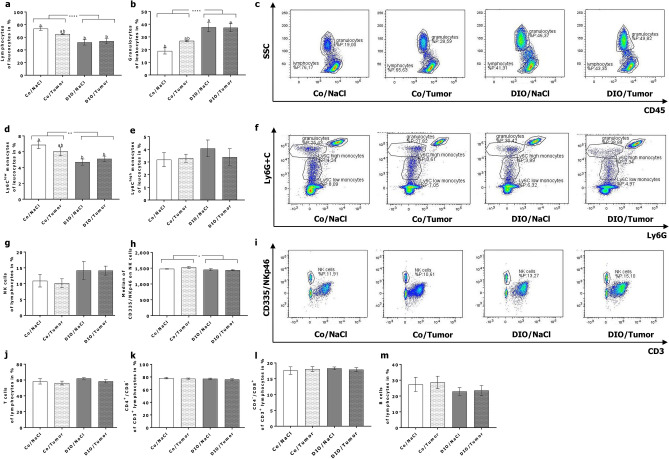
Flow cytometry analysis of peripheral blood immune cells of mice in short-term experiment. Exemplary flow cytometry plots and frequencies of different leukocyte and lymphocyte populations in peripheral blood of control diet-fed (Co) and DIO diet-fed (DIO) mice injected with sodium chloride (NaCl) or 4T1-luc2 breast cancer cells (Tumor). Frequency of lymphocytes (**a**), granulocytes (**b**) and Ly6C^low^ (**d**) and Ly6C^high^ (**e**) monocytes are presented as percentage of leucocytes. Total NK cells (**g**), T cells (**j**), CD4^+^ T cells (**k**), CD8^+^ T cells (**l**), and B cells (**m**) are presented as percentage of lymphocytes. Relative expression of CD335 on NK cells is presented as median fluorescence intensity (Median) (**h**). Values represent means ± SEM, n = 7 mice/group. * indicates significant differences of means between mice receiving DIO diet (DIO) compared to mice receiving control diet (Co) analyzed by two-way ANOVA (* p ≤ 0.05; **** p ≤ 0.0001). Different superscript letters (a,b) indicate significant differences between individual experimental groups analyzed by Tukey´s multiple comparison test (p ≤ 0.05). Differences in frequencies of lymphocytes (**c**), granulocytes (**c**), Ly6C monocytes (**f**) and NK cells (**i**) in mice are exemplary demonstrated.

In the short term experiment two-way ANOVA analysis demonstrated that the frequency of lymphocytes in groups fed the high-fat diet was significantly decreased compared to mice fed the control diet independently of the injection of tumor cells (Fig. 4a,c). In contrast, frequency of granulocytes increased in groups fed the high-fat diet compared to the control diet and significantly differed from control diet-fed animals without tumor cell challenge (Fig. 4b,c). While frequency of Ly6C^high^ monocytes was not altered, frequency of Ly6C^low^ monocytes was significantly decreased in the DIO groups (Fig. 4d–f). Although the frequency of NK cells, defined by the expression of CD335 and the lack of CD3, did not differ, median fluorescence of CD335/natural cytotoxicity triggering receptor (NCR)1/NKp46 was significantly decreased in high-fat diet groups compared to control diet groups (Fig. 4 g–i). Numbers of T cells, T cell subpopulations as well as B cells remained unchanged between the different groups (Fig. 4j–m).

**Figure 5:**
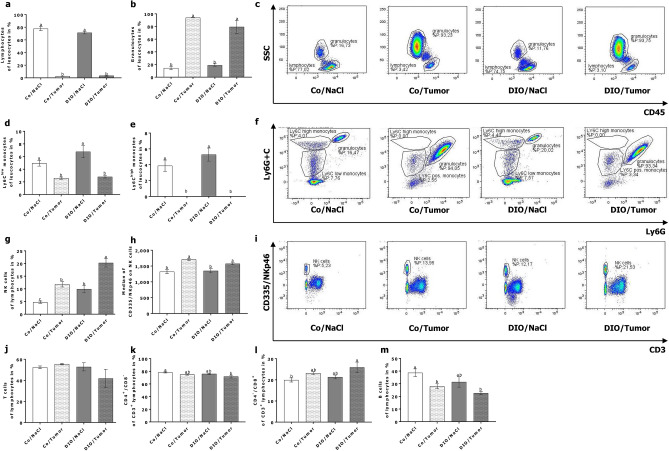
Flow cytometry analysis of peripheral blood immune cells of mice in long-term experiment. Exemplary flow cytometry plots and frequencies of different leukocyte and lymphocyte populations in peripheral blood of control diet-fed (Co) and DIO diet-fed (DIO) mice injcected with sodium chloride (NaCl) or 4T1-luc2 breast cancer cells (Tumor). Frequency of lymphocytes (**a**), granulocytes (**b**) and Ly6C^low^ (**d**) and Ly6C^high^ (**e**) monocytes are presented as percentage of leucocytes. Total NK cells (**g**), T cells (**j**), CD4^+^ T cells (**k**), CD8^+^ T cells (**l**), and B cells (**m**) are presented as percentage of lymphocytes. Relative expression of CD335 on NK cells is presented as median fluorescence intensity (Median) (**h**). Values represent means ± SEM, n = 7 mice/group. Different superscript letters (a,b,c) indicate significant differences between individual experimental groups analyzed by Tukey´s multiple comparison test (p ≤ 0.05). Differences in frequencies of lymphocytes (**c**), granulocytes (**c**), Ly6C monocytes (**f**) and NK cells (**i**) in mice are exemplary demonstrated.

In the long-term experiment mice of tumor groups showed significantly decreased frequency in lymphocytes and Ly6C^low^, while granulocytes were increased significantly (Fig. 5a–f). Moreover, tumor-bearing mice showed a depletion of Ly6C^high^ monocytes in peripheral blood independent of the fed diet (Fig. 5e,f). Percentage of NK cells from lymphocytes were the highest in tumor challenged animals fed the high-fat diet and differed significantly to all other groups (5g, i). Additionally, mice receiving the control diet and the NaCl injection showed the significantly lowest NK cell frequency (Fig. 5 g,i). Two-way ANOVA analysis revealed that the median fluorescence intensity of CD335/NKp46 on NK cells was significantly increased in tumor challenged groups independent of the diet (Fig. 5 h; p ≤ 0.0001). T cell frequency remained unchanged, but two-way ANOVA revealed that tumor growth significantly decreased the frequency of the subpopulation of T helper cells (CD4^+^/CD8^−^; p ≤ 0.05) and increased the frequency of cytotoxic T cells (CD4^−^/CD8^+^; p ≤ 0.05) most pronounced in the DIO/Tumor group (Fig. 5j–l). Frequency of B cells was decreased in tumor challenged mice and differed significantly compared to mice receiving the control diet and NaCl injection (Fig. 5m).

### Immunohistochemically staining and relative mRNA concentrations of NKG2D-receptor and –ligands in adipose tissues of mice

**Figure 6:**
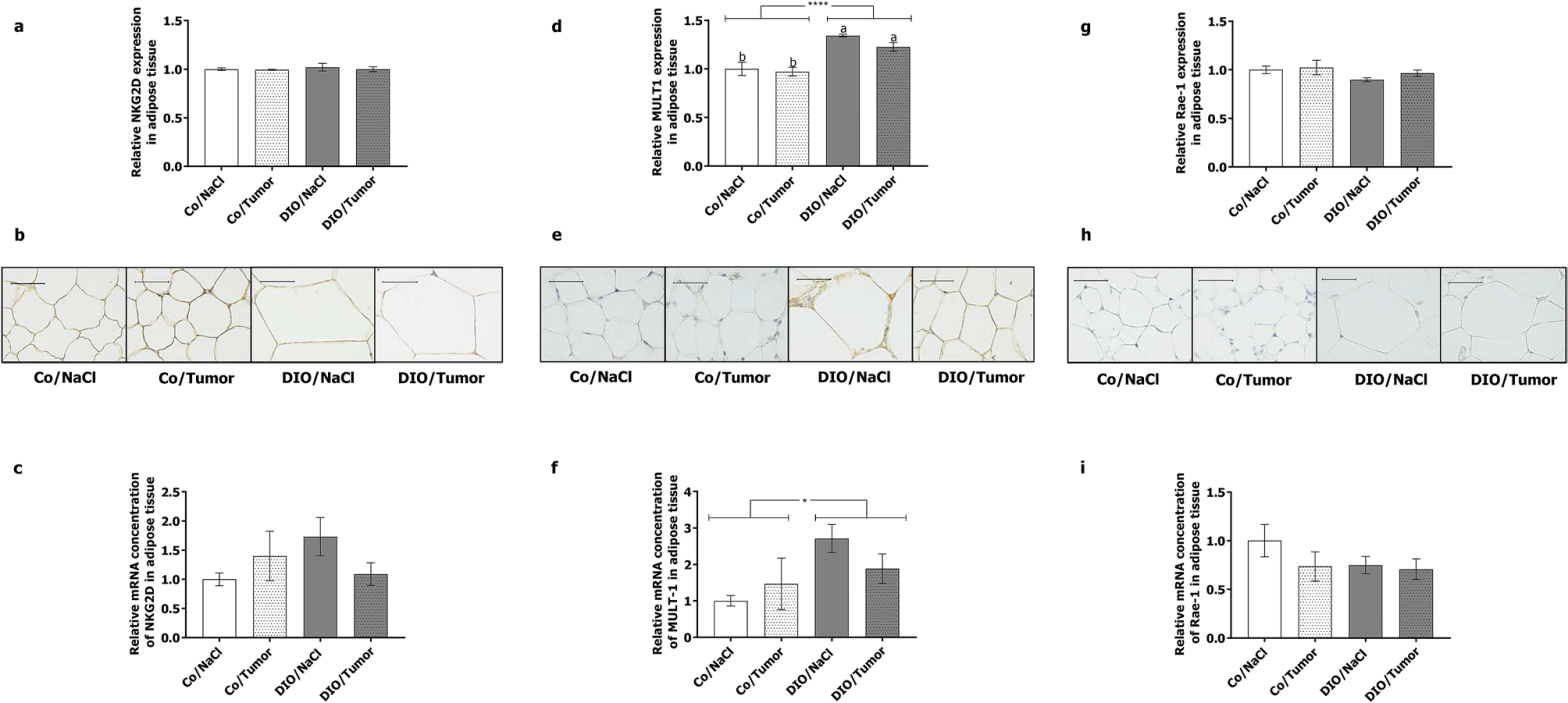
Immunohistochemical staining and expression of NKG2D-receptor and ligands in adipose tissue. Relative expression and representative pictures of immunohistochemically staining of NKG2D-receptor (**a** and **b**); MULT1 (**c** and **d**) and Rae1 (**e** and **f**) in visceral adipose tissues of mice in the long-term experiment receiving either control diet (Co) or DIO diet (DIO) and injection of sodium chloride (NaCl) or 4T1-luc2 breast cancer cells (Tumor). Values represent means ± SEM, n = 5 mice/group and five analysed tissue sections per mice. * indicates significant differences of means between mice receiving DIO diet (DIO) compared to mice receiving control diet (Co) analyzed by two-way ANOVA (**** p ≤ 0.0001). Different superscript letters (**a**,**b**) indicate significant differences between individual experimental groups analyzed by Tukey´s multiple comparison test (p ≤ 0.05). Scale bar (40 µm).

As expected DIO mice showed enlarged, adipocytes compared to control diet-fed groups as demonstrated by representative pictures of adipose tissue of mice in the long-term experiment (Fig. 6b,e,h). To investigate NK cells in adipose tissue, immunohistochemically staining and analysis of the relative mRNA concentration of NKG2D-receptor and the NKG2D–ligands MULT1 and Rae-1 were conducted. Mice fed the high-fat diet showed significantly higher expression of the NKG2D-ligand MULT1 in adipose tissue compared to animals fed the control diet independent of tumor growth (Fig. 6d–f). Expression of NKG2D as well as the corresponding ligand Rae-1 in adipose tissue did not differ between the groups (Fig. 6a–c and g–i).

### Expression of functional NK cell markers on isolated splenic NK cells

**Figure 7:**
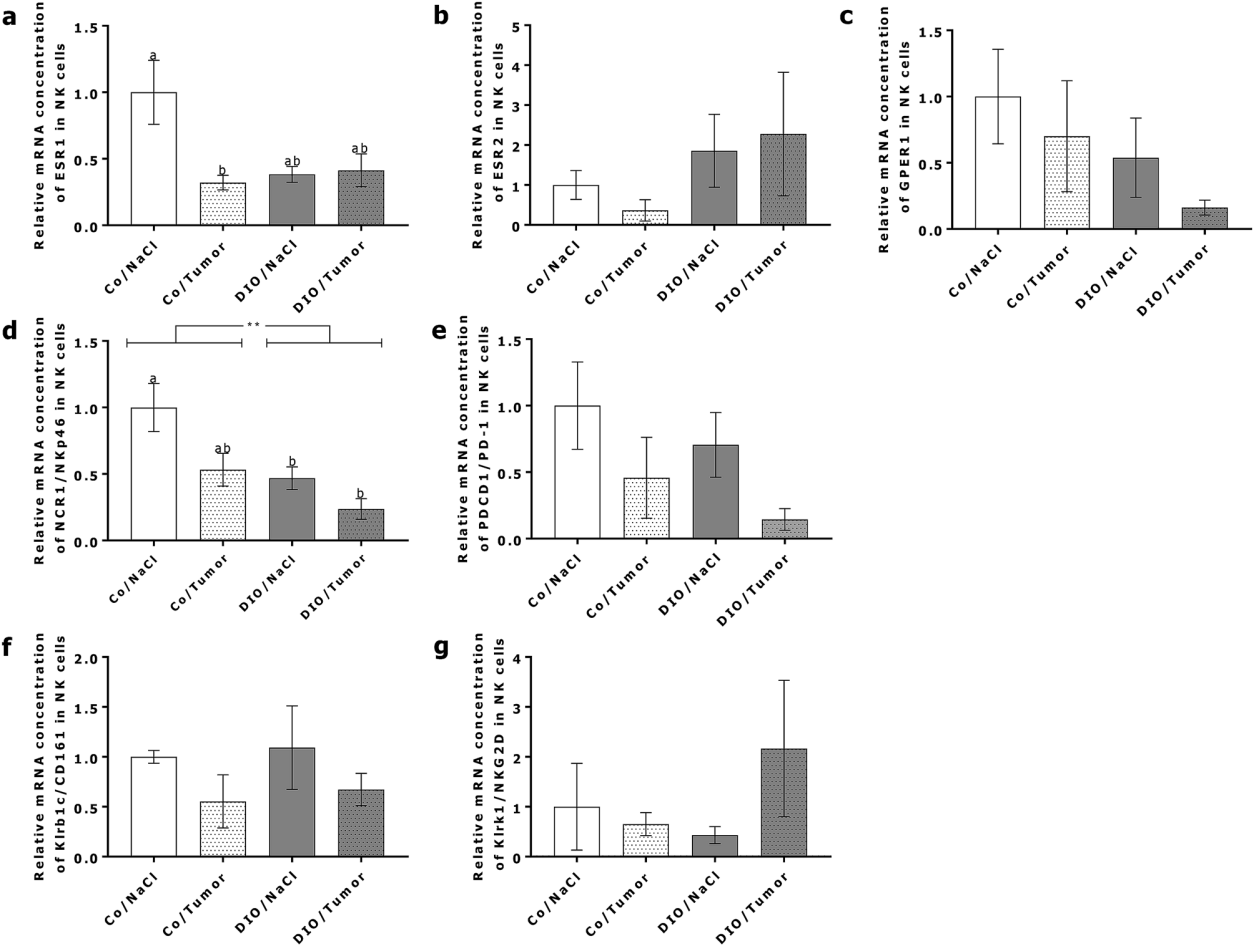
Expression of NK-cell-related markers in splenic NK cells. Relative mRNA concentration of NK-cell-related markers (**a**) ESR1; (**b**) ESR2; (**c**) GPER1; (**d**) NCR1/NKp46; (**e**) PDCD1/PD-1; (**f**) Klrb1c/CD161; (**g**) Klrk1/NKG2D in isolated spleen NK cells of mice in short-term experiment. Values represent means ± SEM, n = 7 mice/group. ** indicates significant differences of means (p ≤ 0.01) between mice receiving DIO diet (DIO) compared to mice receiving control diet (Co) analyzed by two-way ANOVA. Different superscript letters (**a**,**b**) indicate significant differences between individual experimental groups analyzed by Tukey´s multiple comparison test (p ≤ 0.05).

Analysis of the relative mRNA expression of estrogen receptor (ESR) 1 in isolated splenic NK cells of the short-term experiment showed a significantly decreased expression in control diet-fed animals receiving tumor cell injection compared to NaCl-injection. In DIO-fed animals, relative mRNA expression of ESR1 did not differ although they were as low as in mice of the Co/Tumor-group (Fig. 7a). NCR1/NKp46 expression in NK cells was significantly influenced by DIO-diet and tumor cell injection (Fig. 7d). Comparing single groups relative mRNA concentrations of NCR1/NKp46 were significantly decreased in the DIO/NaCl and the DIO/Tumor group compared to animals receiving the control diet and a NaCl-injection (Fig. 7d). Expression of ESR2, G protein-coupled estrogen receptor (GPER) 1, programmed cell death protein 1 (PDCD1/PD-1), killer cell lectin-like receptor subfamily B member (Klrb) 1c/CD161 and Klrk1/NKG2D did not differ between the four experimental groups (Fig. 7b,c,e–g).

## Discussion

Breast cancer is the most common cancer in women worldwide. Approximately 2.1 million diagnoses of breast cancer were estimated for 2018, which accounts for 11.6% of the total cancer incidence burden and results in the leading cause of cancer death in woman^34^. Several risk factors for example aging, nulliparity, family history, menopausal hormone therapy and genetic mutations, have been identified^35^. Nevertheless, also lifestyle factors like diet, nutrition and physical activity, seem to have a great impact on the incidence of breast cancer^36^. In detail, there is a strong evidence that increased body fatness throughout adulthood and adult weight gain are two important risk factors for postmenopausal breast cancer, which is the most diagnosed breast cancer in woman^36^. Mechanisms underlying the risk factor obesity are continuously investigated and the immune system seems to play a central role in the interplay of obesity and the impaired cancer defense^37^. Obesity is associated with a state of chronic low grade inflammation which is represented by a an increased inflammatory profile and an impaired immune regulation^33,38–43^. As NK cells are a central component of the innate immune system and are capable to directly destroy tumor cells^25,44^, the present study aimed to investigate the NK cell phenotype in a diet-induced obese postmenopausal breast cancer mouse model (short-term experiment) complemented by monitoring of tumor-growth (long-term experiment).

As expected, diet-induced obese animals showed significantly higher body weights and visceral fat mass compared to control diet-fed animals. Although tumor development led to loss of body weight in DIO-fed animals and visceral fat mass in both diet groups, significant differences between the diet groups still preserved. Plasma concentrations of leptin and IL-6 were increased in DIO-fed mice after 17 weeks, representing the chronic low grade inflammation of the obese status^45^. This inflammatory condition was underlined by significantly increased frequencies of granulocytes in the mice of the DIO groups, which is in line with results from other studies comparing normal weight and obese subjects^46–48^. To mimic postmenopausal breast cancer, mice were ovariectomized and 4T1-luc2 cells were injected into the fat pad of the mammary gland. This orthotopic and syngeneic breast cancer model in BALB/c mice shares multiple features of the progressive forms of estrogen-insensitive human metastatic breast cancer^49^. Interestingly the final tumor weight did not differ between control and DIO-fed mice. This may be caused by higher appearance of necrosis observed in the tumors of obese mice, resulting in a different density of the solid tumors. Necrosis occurs in fast growing tumors, is a result of inadequate vascularization and is associated with a poor prognosis^50^. Bioluminescence and caliper-based monitoring of tumor growth revealed an increased tumor burden for DIO-fed mice. A study of Kim et al*.* reported similar results, although they used non-ovariectomized obese-resistant BALB/c mice^51^. Hence, it is still a subject of discussion, if the state of obesity per se is causal for the increased cancer incidence or if dietary components like fat are mediating the observed effects^52^.

Interestingly tumor burden of mice in the long-term experiment led to significantly higher spleen and liver weights independent of the diet, which is in accordance with other studies using the 4T1-luc2 cell line for the induction of breast cancer in mice and is based on the elevated hematopoiesis^49,53,54^. This was also seen in markedly increased frequency of granulocytes and decreased frequency of lymphocytes and monocytes in tumor bearing mice of the long-term experiment. In contrast to Trottier et al*.*, who showed an enhanced hematopoiesis in HFD fed mice, we observed significantly decreased lymphocytes in the blood of obese mice compared to control group in the short-term experiment^55^. The chronic low grade inflammation in obesity is associated with an increased infiltration and migration of immune cells into adipose tissue^56^. As higher numbers of lymphocytes and macrophages are found in adipose tissue of obese individuals, it could be assumed that this is causal for the observed decreased frequency of circulating lymphocytes in the present study^57^.

T cells express CD3 and can be further classified by their expression of CD4, representing T-helper cells and CD8, representing cytotoxic T cells. In the long-term experiment, a slight decrease in T-helper cells (CD3^+^/CD4^+^/CD8^−^) and a slight increase in cytotoxic T cells (CD3^+^/CD4^−^/CD8^+^) in peripheral blood was observed in the tumor mice compared to the normal weight mice without tumor cell injection. To our knowledge, until now no data of the frequency of T-cell subsets on DIO-fed tumor-bearing mice exist. One study in rats reported significantly decreased frequencies of CD4^+^ T cells and significantly increased frequencies of CD8^+^ T cells in DIO animals^58^. However, it is known, that in DIO mice and obese humans CD4^+^ and CD8^+^ T cells show an enhanced recruitment and infiltration into the adipose tissue^59–61^. Supposable, the increase of cytotoxic T cell frequency observed in the present study is due to a regulatory response on the increased tumor burden of DIO-fed mice.

High-fat diet was seen to impair B cell-lymphopoiesis of the bone marrow niche and tumor-infiltrating B cells are recognized as a new hallmark in breast cancer^62,63^ . Interestingly, in the present study B cell frequency in the peripheral blood was downregulated in mice of the tumor group as well as by DIO and was the lowest in DIO/Tumor mice. Although not analyzed in the present study, this could be due to the described decreased B cell lymphopoiesies and an increased infiltration of B cells into tumor tissue.

Obesity is associated with increased circulating monocytes^64^, which is found to be linked mechanistically to leptin^65,66^. Mouse monocyte subsets are classified as Ly6C^high^ and Ly6C^low^. Ly6C^high^ monocytes differentiate into Ly6C^low^ monocytes in the circulation. Under obese conditions, Ly6C^high^ monocytes, who represent the inflammatory monocytes, migrate to the side of inflammation and differentiate into adipose tissue macrophages. Circulating Ly6C^low^ monocytes represent the anti-inflammatory monocytes and are resident, patrolling in the blood vessels^67,68^. Interestingly, flow cytometry analyzes in the short-term experiment of the present study revealed a decrease of Ly6C^low^ monocytes in the peripheral blood of DIO mice, while Ly6C^high^ monocytes remained unchanged. However, this effect was diminished in tumor bearing mice as frequency of Ly6C^low^ monocytes was decreased compared to NaCl-injected mice. Ly6C^high^ monocytes even diminished, suggesting that this may be triggered by a higher infiltration of macrophages into the tumor tissue induced by monocyte chemoattractant proteins^69^.

For frequency of NK cells in the peripheral blood of obese individuals controversial data are reported^23,29,33,58,70–72^. In this study NK cell frequencies in the blood of mice were not altered in the short-term experiment. However, in the long-term experiment, DIO led to significantly increased NK cell frequencies and was most pronounced in tumor-challenged DIO-fed mice, probably as a regulatory feedback mechanism. This was accompanied by higher median fluorescence intensity for the activating NK cell receptor CD335/NKp46. Concerning control diet-fed mice and DIO mice bearing the tumor, it shows that although NK cell frequency is higher in the DIO group, the median fluorescence intensity does not differ, suggesting decreased CD335/NKp46-receptor expression per single cell in obese mice. Moreover, this seems to be independent of tumor burden, as this is also true for mice lacking the tumor. This is supported by the fact that relative mRNA expression of CD335/NKp46 in splenic NK cells is significantly decreased by DIO-feeding in the short-term experiment.

The NK cell effector function is mediated by inhibitory and activating surface receptors as well as by adhesion and cytokine receptors^73^. They recognize cell-surface and extracellular-secreted ligands, e.g. major histocompatibility complex class I (MHC-I) and MHC-I-related molecules and non-MHC molecules^74^. MHC-I molecules (human leukocyte antigens (HLAs) in humans and H-2 in mice) are constitutively expressed by nucleated healthy cells and are identified by inhibitory NK cell receptors. The loss of MHC-I upon infection or malignant transformation leads to a predominance of activating signals and therefore to NK cell activation and target cell lysis^75^. Additionally, the expression of activating ligands is necessary for target cell killing and cytokine production by NK cells. Activating ligands are expressed by stressed cells upon infection or cellular transformation^73,76^. Thus, the balance and imbalance between activating and inhibitory signals dynamically regulate the effector function of the NK cell^25,77^. Interestingly, functional studies demonstrated that an increase in co-inhibitory receptor PD-1 expression on NK cells is associated with a decline in NK cell-mediated tumor defense and with a poorer prognosis in digestive cancers^78^. Another study reported higher levels of PD-1 expression upon stimulation in obese children compared to normal weight counterparts, accompanied by a reduced NK cell-mediated killing of target cells^70^. However, analysis of PD-1 expression in splenic NK cells in the present study revealed no alterations between the different diet and tumor challenged groups. The influence of obesity on the expression of activating and inhibiting NK cell receptors is still under debate in the field. Tobin et al*.* and Theurich et al*.* reported no differences in the expression of NK cell receptors in obesity, while studies on rats and humans revealed an impaired expression for NKp46 and NKG2D^58,70–72,79^. As already discussed, in the present study the expression of NKp46 on NK cells was significantly decreased in obese mice. Nevertheless, this was not true for NKG2D and Klrb1c representing additional activating NK cell receptors. Immunohistochemically staining of NKG2D receptor in adipose tissue also revealed no differences between control-fed or DIO-fed mice. As recently reviewed by O´Shea et al*.* NK cells are discussed to regulate adipose tissue homeostasis by killing of inflammatory macrophages after an NK-cell-mediated recruiting to adipose tissue^80^. Wensveen and colleagues hypothesized, that an upregulation of NKp46 ligands in obese adipose tissue leads to an activation of NK cells, which is followed by an IFNγ-induced differentiation of M2 macrophages into inflammatory M1-macrophages^81^. However, in contrast to the present study and results from Chung et al*.*^82^ no upregulation of NKG2D ligands in obese adipose tissue was found. In the present study we cannot clearly proof whether the observed upregulation of NKG2D ligand MULT1 in obese adipose tissue is due to “stressed” fat cells or caused by inflammatory macrophages, which also express NKG2D ligands, therefore making them a target for NK cell lysis^83^. Nevertheless, it can be hypothesized that NK cells are collected by the inflammatory adipose tissue under obesity by upregulation of NKG2D ligand MULT1 and therefore not available for cancer cell killing in tumor tissue. This may have at least in part contributed to the observed increased tumor development in obese mice.

Data about expression of estrogen receptors on NK cells are very limited, which is nicely reviewed by Kovats et al*.*^84,85^. In obesity, ESR on breast cancer cells is downregulated^86,87^. In the present study we chose 4T1-luc2 cells, which are ESR-negative, to induce breast cancer in mice. In postmenopausal obesity estrogen levels are known to be elevated due to higher aromatase activity in peripheral tissues, mainly in adipose tissue^88^. We therefore generated a model to study the effect of higher estrogen levels in a postmenopausal status on NK cells, independent from the direct estrogen-effect on the tumor. It is well established that estrogens decrease NK cell cytotoxicity^84,89–95^ and high estrogen levels lead, as a feedback mechanism, to a downregulation of estrogen receptor expression in breast cancer cells^96,97^. Interestingly in our study, DIO-fed mice tended and tumor bearing control diet-fed mice had significantly decreased expression of ESR1 on NK cells, suggesting a direct regulation of NK cells via increased estrogen levels in postmenopausal breast cancer and obesity. Nevertheless, estrogens are also known to affect tumor growth by the influence of tumor microenvironment and angiogenesis of tumors^98,99^. Therefore, we can only speculate and future studies have to confirm these results, especially based on protein expression.

To conclude, results of the present study describe an altered expression of NK cell receptors and ligands and an increased tumor burden in postmenopausal obese mice. As obesity is a preventable risk factor, it is important to analyze mechanisms underlying the altered NK cell defense of tumor cells by future studies. Recent studies on postmenopausal breast cancer survivors investigating the impact of weight reduction by physical activity or dietetic interventions on the progression of breast cancer^100–103^ highlight the importance of this research field.

## Methods

### Animals and experimental setup

Fifty-six female, ten weeks old, BALB/c mice were purchased from Charles River (Sulzfeld, Germany) and were housed in groups of two in standard type II polycarbonate individual ventilated cages (IVC). Cages were provided with embedding material (LINOCREL FS 14, Altromin, Lage, Germany) and a polycarbonate based mouse igloo (Zoonlab, Castrop-Rauxel, Germany). Mice were maintained at an ambient temperature of 22 ± 2 °C and relative humidity of 65 ± 5% on a 12:12 h light dark cycle with lights on from 6 am to 6 pm. Rodent standard diet (Altromin 1324) and water were available ad libitum*.* After one week of settling in and randomly assignment by body weight, twenty-eight mice received a high-fat diet (50% fat) ad libitum to initiate diet-induced obesity (DIO). Body weight of the mice was detected once per week by an appropriate balance (FTB-BA-d_0720, Kern, Balingen-Frommern, Germany). The federal authorities for animal research in Halle (Germany) approved the experimental protocol. The principles of laboratory animal care were followed according to the guidelines of the European (FELASA) and German Society of Laboratory Animal Sciences (GV-SOLAS). Mice were fed 13–14 weeks with the specific diet until ovariectomy was performed (for details see below). In the following three weeks, mice were allowed to recover from surgery. To induce a mammary carcinoma, half of the animals per diet group were injected 4T1-luc2 cells, which is described in detail below. Sodiumchlorid (NaCl) served as a control. Consequently, four experimental groups (n = 7) resulted: Co/NaCl; Co/Tumor; DIO/NaCl and DIO/Tumor. To enable observation of a short tumor cell challenge (20 h) vs. a long tumor cell challenge (four weeks), two time points of scarification were chosen (Fig. 1a,b).

### Ovariectomy

For anesthesia during ovariectomy a combination of ketamine (Ketavet 100 mg/mL, Zoetis Germany GmbH, Berlin, Germany) and medetomidine (Dorbene vet 1 mg/mL, Zoetis Germany GmbH) was used. Ketamine and medetomidine were suspended in physiological saline solution and injected *i.p.* in a final concentration of 100 mg/kg body weight for ketamine and 1.2 mg/kg body weight for medetomidine. To protect eyes of the mice against dehydration during the surgery, they were moisten with dexpanthenole (Bepanthen 10 g, Bayer AG, Leverkusen, Germany). To further support analgesia, ten minutes after beginning of anesthesia metamizol (Novaminsulfon-ratiopharm 1 g/2 mL, Ratiopharm AG, Ulm, Germany) was applied subcutaneously (lean mice: 10 mg; obese mice: 20 mg). Surgical intervention and awaking period was performed on a warming blanket to prevent mice from cooling. An approximately 0.5 cm long incision through the skin and two incisions through muscle and peritoneum bilaterally and parallel to the backbone were made while mice were placed in prone position. Ovaries were positioned outside the body via these incisions and removed by thermal cautery. Hereafter, incisions were closed using an sterile absorbable thread for in situ suture (V2130H, Ethicon, Norderstedt, Germany) and a sterile synthetic non-absorbable thread for skin suture (EH7147H; Ethicon). For pain management drinking water of the mice was supplemented with 1.6 mg/mL metamizole (Novaminsulfon-ratiopharm 500 mg/mL, Ratiopharm AG) 10 days and the drinking volume was controlled daily.

### Culture and application of tumor cells

Tumor induction in mice was achieved by 4T1-luc2 cell application (Perkin-Elmer; Berlin, Germany) cultured before in vitro as recommended by the manufacturers. This cell line is derived from BALB/cfC3H and expresses luciferase encoded by luc2 gene. It is an established animal model to mimic human breast cancer stage IV and allows bioluminescence monitoring of tumor growth^49^. After induction of DIO and ovariectomy (see above), animals of the tumor groups were injected with 10^6^ 4T1-luc2 cells dissolved in 0.1 ml cell culture medium (RPMI 1640; Gibco, Invitrogen, Carlsbad, California, USA) orthotopically in the subcutaneous fat pad of the fourth right mammary gland under light isoflurane anesthesia as described above. Control animals received 0.1 ml 0.9% NaCl in the same anatomical site.

### Bioluminescence imaging and caliper measurement of tumor growth in the long-term experiment

In the long-term experiment, tumor volume was recorded once per week by caliper measurement using the following formula: length  ×  width^2^ × 0.52. Bioluminescence images were taken on day 7, 14 and 21 after injection of 4T1-luc2 tumor cells. Mice were anaesthetized by light isoflurane inhalation and images were taken exactly 30 min after *i.p.* injection of 150 µl luciferin (PerkinElmer, Massachusetts, USA) and by using VisiLuxII (Visitron Systems GmbH, Puchheim, Germany). Images were analyzed using the FIJII-software^104^.

### Blood and organ sampling

Mice were sacrificed under general isoflurane anesthesia either after 20 h (short-term experiment) or four weeks (long-term experiment) by puncture of the right heart ventricle. Blood was withdrawn and spleen, visceral adipose tissue, liver and tumors were resected. Organs were weighed and immediately frozen in liquid nitrogen and stored at − 80 °C for RNA isolation and lipid analysis. Adipose tissue was formalin fixed and paraffin embedded. Blood samples were either used for plasma collection or stored on crushed ice for cytometry analyzes.

### Immunoassay of plasma samples

Plasma concentrations of the (adipo)cytokines leptin, TNF-α, IL-6 and IFN-γ were quantified using a multiplex immunoassay (eBioscience, Thermo Fisher Scientific Inc., Waltham, USA) following the manufacturer's instructions. Data were analyzed using the Procartaplex-analyst 1.0 software (eBioscience).

### Immunhistochemical stainings of adipose tissue

Paraffin sections (5 μm) of visceral adipose tissue were prepared and stained with the mAbs anti-NKG2D (ab203353, Abcam, Cambridge, United Kingdom) and anti-UL16 Binding Protein 1 (ULBP1/MULT1, ABIN966609, antibodies online, Aachen, Germany) using the EnVision + System (horseradish peroxidase (HRP) labelled polymer anti-rabbit, Dako North America Inc., Carpinteria, USA). For Rae-1 staining mAB Rae-1 pan specific antibody (AF1136, R&D Systems, Minneapolis, USA) and anti-Goat HRP-DAB Cell & Tissue Staining Kit (CTS008, R&D Systems) were used. For both staining protocols, 3,3′-diaminobenzidine (DAB) was used as a chromogen for visualization of the respective proteins. Finally, sections were counterstained with hematoxylin and covered with Eukitt mounting medium (Sigma Aldrich, St. Louis, Missouri, USA). In the following, five tissue sections per mouse (n = 5) were analyzed with unknown group identity using a microscope (BZ9000 Fluorescence Microscope, Keyence, Neu-Isenburg, Germany) and FIJII-software with colour deconvolution plugin^104^. Per random offset function a grid of squares with the size of 12 × 10^8^ µm^2^ was created and eight individual fields were used to determine DAB-positive and hematoxylin-positive pixels, excluding the following criteria: a) blood vessel; b) artefacts, e.g. air bubbles, blur, spots; c) damaged tissue; d) neighboring fields. The relative expression of the respective proteins was calculated by the formula: *pixel(DAB)/(pixel(DAB)* + *pixel (hematoxylin)),* according to Shindhu et al*.*^105^. Hereby, the influence of obesity-related hypertrophy of adipose tissue could be taken into account and was equalized.

### Flow cytometry analysis

For flow cytometry analysis, whole blood samples were stained following the almost identical protocol already described elsewhere^72^ by using the following fluorochrome-labeled anti-mouse mAbs: FITC Rat Anti-Mouse CD45; APC Rat Anti-Mouse Ly-6G; PE-CF594 Rat Anti-Mouse Ly-6G and Ly-6C; PE-Cy7 Rat Anti-Mouse CD4; Alexa Fluor 700 Rat Anti-Mouse CD8a; APC-Cy7 Rat Anti-Mouse CD45R; PE Hamster Anti-Mouse CD3e and PerCP-Cy5.5 Rat Anti-Mouse CD335 (BD Biosciences, San Diego, USA). In brief, protected from light, blood was incubated for 15 min at room temperature. The stained samples were then treated with BD FACS lysing solution (BD Biosciences) following manufacturer’s instructions. Thereafter, white blood cells were washed twice with washing buffer (PBS, 1% BSA and 0.1% sodium azide), resuspended in measuring buffer (PBS, 0.1% BSA and 0.1% sodium azide) and measured by flow cytometry using LSR Fortessa (BD Biosciences, San Diego, USA). Plots were compensated and analyzed by FlowLogic Software 7.2.1 (Inivai Technologie, Mentone Victoria, Australia). Gating strategy is shown in Supplemental Fig. 1.

### Realtime RT-PCR analysis

Splenic NK cells were directly isolated by magnetic activated cell sorting (MACS) using an NK cell isolation kit (Miltenyi, Bergisch Gladbach, Germany). Thereafter, mRNA was isolated by using Dynabeads oligo (dT) (Invitrogen, Thermo Fisher Scientific Inc.) following manufacturer´s instructions. Synthesis of cDNA was performed by reverse transcriptase reaction according to the supplier's instruction as already described (Thermo Fisher Scientific Inc.) as already described^72^. The mRNA concentrations of genes were measured by real-time polymerase chain reactions (qTower, Analytik Jena AG, Jena, Germany) using SYBR Green Fluorescein Mix (BioRad, München, Germany) and the specific primers (KiCq- Start Primers, Sigma Aldrich, Supplementary Table S3). For normalization of target gene values, the housekeeping gene peptidylprolyl isomerase A (Ppia) was used. The relative mRNA concentration was calculated using the ΔΔCt method and individual amplification efficiency for each primer, determined by a standard curve with different primer dilutions^106^. The mRNA concentrations of genes were measured by realtime detection reverse transcriptase-PCR (iQ5, BioRad) using SYBR Green MIX (BioRad). For determination of mRNA concentration, a threshold cycle (Ct) was obtained from each amplification curve using the software qPCRsoft 3.4 (Analytik Jena AG).

### Statistical analysis

Data analysis was performed using Graph Pad Prism software V7 (GraphPad Inc., La Jolla, CA, USA). Analyzes were performed as two-way ANOVA for the two main factors “diet” and “tumor” followed by Tukey´s multiple comparison test, for individual comparison of all groups. Results are presented as means ± standard error of the mean (SEM). Means were considered as significantly different at p ≤ 0.05. * indicates significant differences between mice receiving the DIO diet compared to control diet. Different superscript letters (a,b,c) indicate significant differences between individual experimental groups.

## Supplementary information

Supplementary Information.

## Data Availability

All data generated or analyzed during this study are included in this published article and its additional files.
