# Supplementary material for: Effects of obesity on NK cells in a mouse model of postmenopausal breast cancer
Source: Sci Rep. 2020 Nov 26;10:20606. doi: 10.1038/s41598-020-76906-5 (PMC7692502; doi:10.1038/s41598-020-76906-5)
Supplement: Supplementary file 1 — Supplementary Information. [file 41598_2020_76906_MOESM1_ESM.pdf]

Effects of obesity on NK cells in a mouse model of postmenopausal breast cancer

Julia Spielmann[1]*, Laura Mattheis[1,2], Juliane-Susanne Jung[1], Henrik Rauße[1,3], Markus Glaß[4], Ina Bähr[1], Dagmar Quandt[1,5], Jana Oswald[1], Heike Kielstein[1]

[1]Institute of Anatomy and Cell Biology, Medical Faculty of Martin Luther University Halle-Wittenberg, Halle (Saale), Germany

[2] Deptartment of Internal Medicine I, Medical Faculty of Martin-Luther University Halle-Wittenberg, Halle (Saale), Germany [3]Clinic for Psychosomatics and Psychotherapy, Landschaftsverband Westfalen-Lippe Clinic, Lengerich, Germany

[4]Institute of Molecular Medicine, Charles Tanford Protein Center, Medical Faculty of Martin Luther University Halle-Wittenberg, Halle (Saale), Germany

[5]Regenerative Medicine Institute (REMEDI) at CÚRAM Centre for Research in Medical Devices, School of Medicine, College of Medicine, Nursing and Health Sciences, National University of Ireland Galway, Ireland

Corresponding author:

*Julia Spielmann

Institute of Anatomy and Cell Biology

Martin Luther University Halle-Wittenberg, Faculty of Medicine

Grosse Steinstrasse 52

06108 Halle (Saale)

Germany

Email: Julia.Spielmann@medizin.uni-halle.de

Supplementary Table S1: Body and organ weight in short-term experiment

	Short-term experiment						
**Time point**	**20h after 4T1-luc2 injection**						
	**Co/NaCl**	**Co/Tumor**	**DIO/NaCl**	**DIO/Tumor**	**Two way ANOVA p-value**		
					**Interaction**	**Tumor**	**Diet**
Body weight in g							
Start of experiment	22.4 ± 0.5	23.0 ± 0.6	22.0 ± 0.8	22.6 ± 0.3	0.9694	0.2831	0.4687
End of experiment	25.5 ± 0.3[b]	25.7 ± 0.3[b]	30.6 ± 1.1[a]	30.6 ± 0.5[a]	0.8015	0.8870	**<0.0001**
Visceral fat in g	0.5 ± 0.05[b]	0.57 ± 0.04[b]	2.02 ± 0.30[a]	2.05 ± 0.19[b]	0.9167	0.7965	**<0.0001**
Spleen weight in g	0.13 ± 0.01[ab]	0.12 ± 0.01[b]	0.15 ± 0.01[ab]	0.17 ± 0.02[a]	0.3029	0.6395	**0.0041**
Liver weight in g	1.15 ± 0.03	1.08 ± 0.01	1.16 ± 0.05	1.16 ± 0.03	0.3443	0.4126	0.1824

Values represent means ± SEM, n=7 mice/group. Bold p-values highlight significant results analyzed by Two-way ANOVA ($p \leq 0.05$). Different superscript letters (a,b) indicate significant differences between individual experimental groups analyzed by Tukey´s multiple comparison test ($p \leq 0.05$).

Supplementary Table S2: Body and organ weight in long-term experiment

	Long-term experiment						
**Time point**	**21 days after 4T1-luc2 injection**						
	**Co/NaCl**	**Co/Tumor**	**DIO/NaCl**	**DIO/Tumor**	**Two way ANOVA p-value**		
					**Interaction**	**Tumor**	**Diet**
Body weight in g							
Start of experiment	22.0 ± 0.5	20.4 ± 0.6	20.9 ± 0.2	21.1 ± 0.5	0.1038	0.2073	0.7561
End of experiment	27.4 ± 0.8 [c]	25.8 ± 0.5 [c]	37.8 ± 1.2 [a]	31.8 ± 1.1 [b]	**0.0223**	**0.0003**	**<0.0001**
Visceral fat in g	0.71 ± 0.05 [b]	0.17 ± 0.04 [c]	2.4 ± 0.20 [a]	0.53 ± 0.10 [bc]	**<0.0001**	**<0.0001**	**<0.0001**
Spleen weight in g	0.12 ± 0.01 [a]	1.28 ± 0.08 [b]	0.15 ± 0.02 [a]	1.44 ± 0.09 [b]	0.3965	**<0.0001**	0.1876
Liver weight in g	1.15 ± 0.04 [b]	1.55 ± 0.06 [a]	1.55 ± 0.07 [a]	1.66 ± 0.07 [a]	**0.0416**	**0.0008**	**0.0008**

Values represent means ± SEM, n=7 mice/group. Bold p-values highlight significant results analyzed by Two-way ANOVA ($p \leq 0.05$). Different superscript letters (a,b,c) indicate significant differences between individual experimental groups analyzed by Tukey´s multiple comparison test ($p \leq 0.05$).

Supplementary Table S3. Characteristics of the specific primers used for real-time RT-PCR analysis

Gene	Forward and reversed primers	bp	Annealing temperature	NCBI gene bank
**Ppia**	for: 5’ CACCGTGTTCTTCGACATCA 3’<br>rev: 5’ TGTCTGCAAACAGCTCGAAG 3’	71	64°C	NM_008907.1
**Klrb1c/CD161**	for: 5’ AACTGAGATTCCTACTGGAC 3’<br>rev: 5’ TTGTGCCATTTATCCACTTC 3’	105	60°C	NM_001159904
**Klrk1/NKG2D**	for: 5’ AGTATTGTGCAACAAGGAAG 3’<br>rev: 5’ TTTTGAGACAACCAGGAAGC 3’	153	57°C	NM_033078.4
**CD335/NCR1/NKp46**	for: 5’ TAGTAACTGGTCTGTATGACAC 3’<br>rev: 5’ CTTGAGCAGAAAGAATTTGC 3’	125	58°C	NM_010746
**PDCD1/PD-1**	for: 5’ ACTAGGGCAATAAAGGGAAC 3’<br>rev: 5’ GAATGAGGAGATTCTAACACC 3’	176	57°C	NM_008798
**ESR1**	for: 5’ CAAGGTAAATGTGTGGAAGG 3’<br>rev: 5’ GTGTACACTCCGGAATTAAG 3’	140	58°C	NM_007956.5
**ESR2**	for: 5’ ATGCTTCTTTCTCATGTCAG 3’<br>rev: 5’ TCTTTGCTCTTACTGTCCTC 3’	188	58°C	NM_010157.3
**GPER1**	for: 5’ GATCTAGGGAGAAAGCCATC 3’<br>rev: 5’ CCTGTTAGTCTCAGAAAACC 5’	181	60°C	NM_029771

RT-PCR, Reverse transcription polymerase chain reaction; Bp, base pair; NCBI, National Center for Biotechnology Information; Ppia, peptidylprolyl isomerase A; Klrb1c/CD161, Killer cell lectin-like receptor subfamily B member 1c; Klrk1/NKG2D, Killer cell lectin-like receptor subfamily k member/ Natural Killer group 2 member D; CD335/NCR1/NKp46, natural cytotoxicity triggering receptor 1; PDCD1/PD-1, programmed cell death protein 1; ESR, estrogen receptor; GPER1, G protein-coupled estrogen receptor 1.

Supplementary Figure 1: Gating strategy for FACS analysis

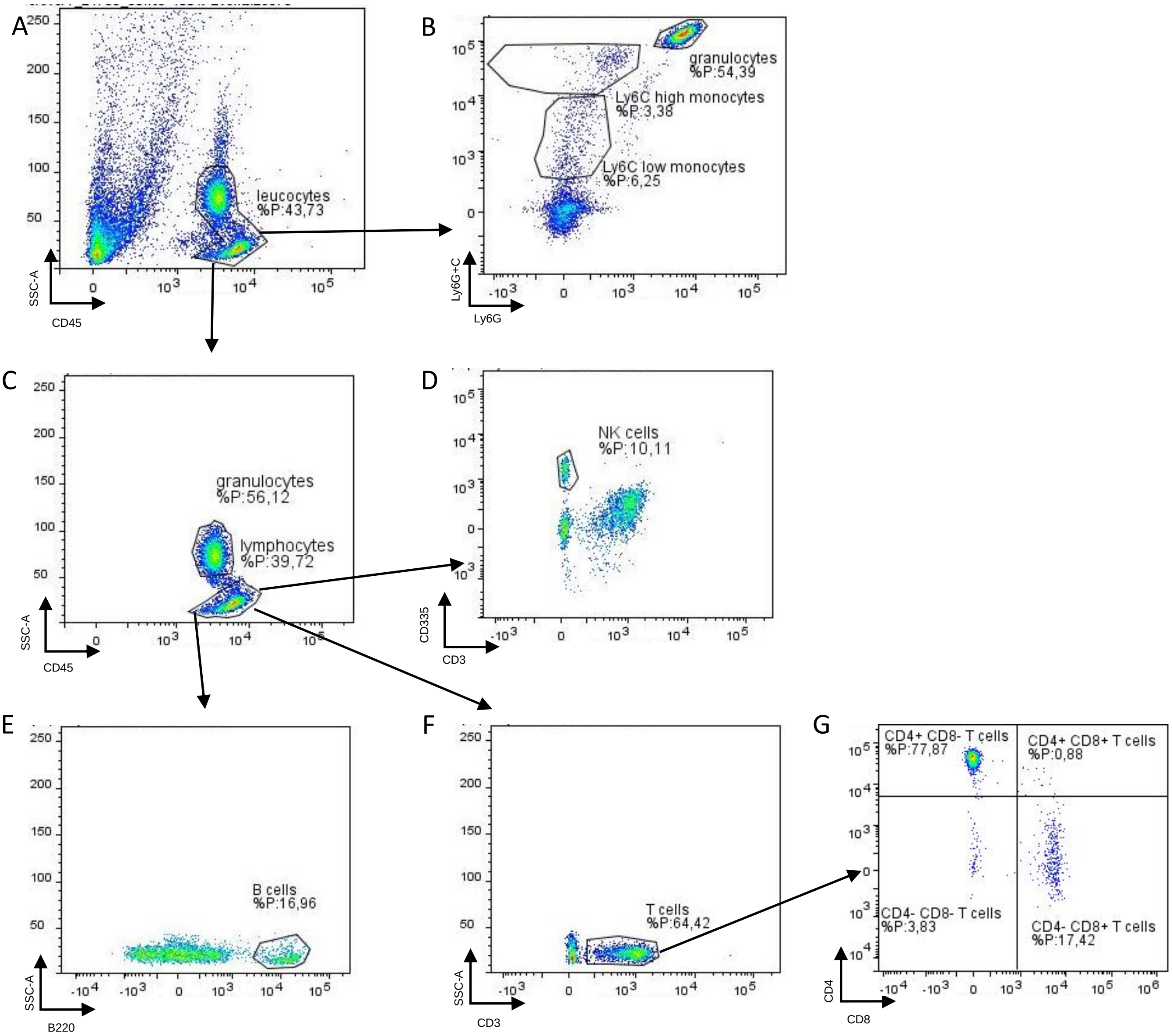

Representative plots of the hierarchical gating strategy to identify immune cell populations and NK cells in BALB/c mice. Leucocytes were identified by their CD45 expression and granularity using SSC (A), followed by gating of $CD45^+$ SSC^{low} lymphocytes and $CD45^+$ SSC^{high} granulocytes (C). Leucocyte gating combined with single cell gating of Ly6G+C and Ly6C expression leads to the identification of $Ly6C^{high}$ monocytes and $Ly6C^{low}$ monocytes (B). Based on the lymphocyte gate, $CD3^-$ and $CD335^+$ lymphocytes were identified as NK cells (D), $B220^+$ SSC^{low} lymphocytes were identified as B cells (E) and $CD3^+$ SSC^{low} lymphocytes were identified as T cells (F). Based on the T cell gate, $CD4^+/CD8^-$ and $CD4^-/CD8^+$ cells were gated and identified as T cell subsets (G).
